# Reviewer acknowledgement 2012

**DOI:** 10.1186/1546-0096-11-8

**Published:** 2013-04-12

**Authors:** Alberto Martini, Charles Spencer

**Affiliations:** 1 University of Genoa, Genoa, Italy; 2 Ohio State University, Columbus, USA

## Abstract

**Contributing reviewers:**

The Editors of *Pediatric Rheumatology* would like to thank all our reviewers who have contributed to the journal in Volume 10 (2012).

## 

Judith Adams

United Kingdom

Kazunaga Agematsu

Japan

Amita Aggarwal

India

Jonathan Akikusa

Australia

Graciela Alarcon

United States of America

Florence Allantaz

United States of America

Georgina Marian Allen

United Kingdom

Meloni Antonella

Jordan

Tadej Avcin

Slovenia

Noha Azab

Egypt

Gerard Banez

United States of America

Mara Becker

United States of America

Edward Behrens

United States of America

Susanne Benseler

Canada

Timothy Beukelman

United States of America

John Bohnsack

United States of America

Zulkif Bozgeyik

Turkey

Riva Brik

Israel

Maggie Bromberg

United States of America

Ruben Burgos-Vargas

Mexico

David Cabral

Canada

Hans-Dieter Carl

Germany

Magda Carneiro-Sampaio

Brazil

Peter Chira

United States of America

Rolando Cimaz

Italy

Mark Connelly

United States of America

Randy Cron

United States of America

Yanick Crow

United Kingdom

Megan Curran

United States of America

Bo Ding

Sweden

Jie Ding

China

Debbie Ehrmann Feldman

Canada

Andrew Eichenfield

United States of America

Nahla El Sharkawy

Egypt

Michelle Ernst

United States of America

Subhadra Evans

United States of America

Corona Fabrizia

Italy

Fernanda Falcini

Italy

Tzipora Falik-Zaccai

Israel

Flavio Fantini

Italy

Anders Fasth

Sweden

Brian Feldman

Canada

Polly Ferguson

United States of America

Natalya Fish

United States of America

Berit Flatø

Norway

Ivan Foeldvari

Germany

Leng Huat Foo

Malaysia

Antonella Forlino

Italy

Helen Foster

United Kingdom

Joost Frenkel

Netherlands

Abraham Gedalia

United States of America

Stuart Goodman

United States of America

Rakesh K Gupta

India

Kathleen Haines

United States of America

Keri Hainsworth

United States of America

Liora Harel

Israel

Troels Herlin

Denmark

Kristin Houghton

Canada

Len Hua

United States of America

Zeng Huasong

China

Kimme Hyrich

United Kingdom

Olcay Jones

United States of America

Ludmyla Kachko

Israel

Philip Kahn

United States of America

Joseph Kahwaji

United States of America

George Kalliolias

United States of America

Sylvia Kamphuis

Netherlands

Ozgur Kasapcopur

Turkey

Raju Khubchandani

India

Na Rae Kim

South Korea

Yukiko Kimura

United States of America

Marisa Klein-Gitelman

United States of America

Susan Klepper

United States of America

Ichiro Kobayashi

Japan

Bianca Lang

Canada

Mario Lapecorella

Italy

Ronald Laxer

Canada

Martine Le Merrer

France

Thomas J A Lehman

United States of America

Carol Lindsley

United States of America

Gustaf Ljungman

Sweden

Deirdre Logan

United States of America

Kathleen Loomes

United States of America

Silvia Magni-Manzoni

Italy

Outi Makitie

Finland

Clara Malattia

Italy

Alberto Martini

Italy

Janet McDonagh

United Kingdom

Hans-Joachim Mentzel

Germany

Kirsten Minden

Germany

Carlomaurizio Montecucco

Italy

Terry Moore

United States of America

Lakshmi Moorthy

United States of America

Zoilo Morel Ayala

Paraguay

Timothy Niewold

United States of America

Peter Nigrovic

United States of America

Megumi Okumura

United States of America

Sheila Oliveira

Brazil

João Oliveira

United States of America

Karen Onel

United States of America

Ingrid Landgraff Östlie

Norway

Haluk H Oztekin

Turkey

Lauren Pachman

United States of America

Murray Passo

United States of America

Anjali Patwardhan

United States of America

Stephen Popper

United States of America

Michael Portman

United States of America

Sampath Prahalad

United States of America

Lynn Punaro

United States of America

Egla Rabinovich

United States of America

Athimalaipet Ramanan

United Kingdom

Angelo Ravelli

Italy

Sarah Ringold

United States of America

Carlos Rose

United States of America

Kelly Rouster-Stevens

United States of America

Ricardo Russo

Argentina

Tomoyuki Saito

Japan

Rotraud Saurenmann

Switzerland

Ken Schikler

United States of America

Qing Shang

Hong Kong

David Sherry

United States of America

Surjit Singh

India

Elisabeth Solau-Gervais

France

Steven Spalding

United States of America

Francois Spertini

Switzerland

Matthew Stoll

United States of America

Jau-Ling Suen

Taiwan

Andrea Superti-Furga

Switzerland

Joost Swart

Netherlands

Yilmaz Tabel

Turkey

Tim Takken

Netherlands

Lene Terslev

Denmark

Andrea Tesija Kuna

Croatia

Wendy Thomson

United Kingdom

Shirley Tse

Canada

Marinka Twilt

Canada

Nikolay Tzaribachev

Germany

Janjaaap van der Net

Netherlands

Sebastiaan Vastert

Netherlands

James Verbsky

United States of America

Linda Wagner-Weiner

United States of America

Lucy Wedderburn

United Kingdom

Patience White

United States of America

Christer Wingren

Sweden

Carine Wouters

Bermuda

Nico Wulffraat

Netherlands

Swan Sim Yeap

Malaysia

Andrew Zeft

United States of America

Xuan Zhang

China

Aristeidis Zibis

Greece

